# Optimal Information Update for Energy Harvesting Sensor with Reliable Backup Energy

**DOI:** 10.3390/e24070961

**Published:** 2022-07-11

**Authors:** Lixin Wang, Fuzhou Peng, Xiang Chen, Shidong Zhou

**Affiliations:** 1 Department of Electronic Engineering, Tsinghua University, Beijing 100084, China; wanglx19@mails.tsinghua.edu.cn; 2 School of Electronics and Information Technology, Sun Yat-sen University, Guangzhou 510006, China; pengfzh@mail2.sysu.edu.cn (F.P.); chenxiang@mail.sysu.edu.cn (X.C.)

**Keywords:** age of information, information update, energy harvesting, reliable backup energy

## Abstract

The timely delivery of status information collected from sensors is critical in many real-time applications, e.g., monitoring and control. In this paper, we consider a scenario where a wireless sensor sends updates to the destination over an erasure channel with the supply of harvested energy and reliable backup energy. We adopt the metric age of information (AoI) to measure the timeliness of the received updates at the destination. We aim to find the optimal information updating policy that minimizes the time-average weighted sum of the AoI and the reliable backup energy cost. First, when all the environmental statistics are assumed to be known, the optimal information updating policy exists and is proved to have a threshold structure. Based on this special structure, an algorithm for efficiently computing the optimal policy is proposed. Then, for the unknown environment, a learning-based algorithm is employed to find a near-optimal policy. The simulation results verify the correctness of the theoretical derivation and the effectiveness of the proposed method.

## 1. Introduction

Timely information updates from wireless sensors to destinations are essential for real-time monitoring and control systems. To describe the timeliness of information updates from the receivers’ perspective, a new metric called age of information (AoI) is proposed [1,2,3]. Unlike general performance metrics, such as delay and throughput, AoI refers to the time elapsed since the generation of the latest received information. A lower AoI generally reflects more timely information at the destination. Therefore, the AoI-minimal status updating policies in sensor networks have been widely studied [4,5,6,7].

The destinations always desire information updates in as timely a manner as possible, which is typically constrained by sensors’ energy. Generally, energy sources include the grid and sensors’ own non-rechargeable batteries. We call these sources *reliable energy* since they enable sensors to reliably operate until the power grid is cut off or sensors’ batteries are exhausted [8]. Specifically, if sensors consume energy from the grid, they need to pay the electricity bill; if sensors only use the power of their own batteries, the price of sensing and transmitting updates will be the cost of frequent battery replacement. There is clearly a price to pay for using reliable energy to update. Energy harvesting (EH) is a promising technology that can help reduce the consumption of reliable energy for information update [9,10]. It can continuously extract energy from solar power, ambient RF, and thermal energy and store the harvested energy in sensors’ rechargeable batteries. The stored energy is renewable and can be used for free. Hence, in this case, the reliable energy can serve as *backup* energy. The design of the coexistence of reliable backup energy and harvested energy has been researched and promoted in academia and industry [8,11,12,13,14]. The mixed energy supply mode can enhance the reliability of the system.

However, the irregular arrivals of harvested energy and the limited capacity of rechargeable batteries still motivate us to schedule the energy usage properly to reduce the cost of using reliable backup energy while maintaining the timeliness of information updates (i.e., the average AoI). Intuitively, the average AoI and the cost of using reliable energy cannot be minimized simultaneously. On the one hand, a lower average AoI means that the sensor senses and transmits updates more frequently, which will increase the consumption of reliable backup energy since the harvested energy is limited. On the other hand, to reduce the cost of reliable backup energy, the sensor will only exploit the harvested energy. Due to the uncertainty of the energy harvesting behavior, the average AoI of the system will inevitably increase. Therefore, in this paper, we focus on achieving the best trade-off between the average AoI and the cost of reliable backup energy.

We consider a sensor-based information update system, where an energy harvesting sensor with reliable backup energy sends timely updates to the destination through an erasure channel. Based on our settings, we will minimize the long-term average weighted sum of the AoI and the paid reliable energy cost to find the optimal information updating policy by Markov decision process (MDP) theory [15]. First, we assume that the sensor knows the relevant statistics in advance, such as the success probability of each transmission and the probability of energy arrival, so that the sensor can make the optimal decision at any time. Then we consider a more realistic scenario where the sensor has no knowledge of the environment. In such an unknown environment, learning-based approaches should be adopted to obtain the updating policy.

### 1.1. Related Work

There have been a series of related works studying AoI minimization in EH communication systems [16,17,18,19,20,21,22,23,24,25,26,27,28,29,30,31,32,33,34]. In these systems, each update consumes harvested energy and is constrained by the energy causality.

Refs. [16,17,18,19,20,21,22,23] focus on how to optimize AoI under general energy causality constraints, where different battery model settings are considered. Constrained by the average power available in the infinite-sized battery, ref. [16] shows that a lazy policy which leaves a certain idle period between updates outperforms the greedy policy under random service times. With the same assumption of an infinite-sized battery, ref. [17] focuses on both offline and online policies under energy replenishment constraints with zero service time. While considering fixed service times, the offline results in [17] are extended to a two-hop scenario in [18], and online policy is provided in [19]. In the case of the delay being controlled by transmission energy, ref. [20] also investigated the optimal offline policy. For the error-free and delay-free channel, the optimal updating policies were investigated for different battery settings [21,22]. Ref. [21] derived the asymptotically optimal policies for the infinite-sized, finite-sized, and unit-sized battery by renewal theory. It turned out to be a threshold policy for the unit-sized battery case. More general battery models were considered in [22]. The optimal policy was also proved to be multi-threshold and the energy-dependent thresholds were characterized explicitly. When the battery is finite sized and there is no feedback from the destination, it was shown that the optimal updating policy is of a threshold structure and the threshold is non-increasing with the battery level [23].

Refs. [24,25,26,27,28,29,30] studied how to properly utilize the harvested energy to transmit updates over imperfect channels. For the noisy channel, ref. [24] considered an infinite-sized battery model and derived the different optimal policies for updating with and without feedback. Ref. [25] further derived a closed-form expression for the threshold of the unit-sized battery model and extended the threshold-based policies to multiple sources case. To combat the noisy channel, some channel coding schemes for EH communication were investigated in [26,27]. In [28], the HARQ protocol was applied for a single EH sensor to send updates to the destination. The optimal policies were obtained by employing reinforcement learning in both known and unknown environments, but no clear intuition on the policy structure was provided. Considering energy harvesting wireless sensor networks (EH-WSNs), ref. [29] suggested to estimate the channel state of a Rayleigh fading channel before transmitting to improve the AoI, update interval and packet loss performance, despite the associated time and energy costs. Ref. [30] aimed to minimize the average AoI of an EH-aided secondary user(SU) in a cognitive ratio network. The SU has to make sensing and updating decisions subject to random energy arrivals and the available spectrum. The sequential decision problem is formulated as a partially observable Markov decision process (POMDP).

Refs. [31,32,33,34] paid attention to other AoI-related metrics in EH communication and even the distributional properties of AoI, not just the average AoI. Different freshness metrics were considered, such as nonlinear AoI [31], urgency-aware AoI (U-AoI) [32], and peak AoI [33] in EH sensor network. To better understand the distributional properties of AoI, ref. [34] further derived closed-form expressions of the moment generating function (MGF) of AoI in an EH-powered queuing system using the stochastic hybrid systems (SHS) framework.

The above works focus on optimizing information freshness under the EH supply. Different from them, energy sources in this paper include both harvested energy and reliable backup energy, and our goal is to achieve the best trade-off between age and reliable energy consumption, instead of merely optimizing AoI. Among the above works, refs. [23,25] are the most related to our paper. The following Table 1 summarizes the detailed differences. It is worth noting that by letting the reliable energy consumption be small enough, our results can be compared with some prior results in [23,25].

The age–energy trade-off has been widely studied in [35,36,37,38,39]. The age–energy trade-off in the erasure channel was studied in [35], and the fading channel case was investigated in [36]. Ref. [37] adopted a truncated automatic repeat request (TARQ) scheme and characterized the age–energy trade-off for the IoT monitoring system. Optimum energy efficiency and AoI trade-off was considered in a multicast system in [38]. In [39], the authors investigated the optimal age–energy trade-off, where status sensing and data transmission can be carried out separately. By the MDP analysis similar to [6,15], the optimal policy exists and is proved to have two thresholds. The energy sources are all reliable in these works, which means that the energy cost of the update is easy to track. However, the uncertainty of the energy arrival and mixed energy supplies bring more challenges to the MDP analysis in this paper. To the best of our knowledge, this paper is the first to consider the timeliness of the system under mixed energy supplies. The preliminary results of this paper are presented in [40].

### 1.2. Main Contributions

The main contributions of this paper are as follows:

- We consider an information update system where the harvested energy and reliable energy coexist. The goal is to find the optimal policy that achieves the best trade-off between age and reliable energy consumption. Compared to the existing works [23,25], our problem is more practical and general, which will provide some insights for future green and durable update system designs.
- For the case that all the statistics such as channel erasure probability and EH probability are known a priori, we formulate an unconstrained infinite space Markov decision process (MDP) problem, and prove the existence of the optimal policy. By revealing the monotonicity and *proportional differential property*of the value function, we find that the optimal policy is of the threshold-type. Based on this special structure, we propose an efficient algorithm to compute the optimal policy.
- In an unknown environment, we propose an average cost *Q*-learning algorithm to obtain the updating policy.
- Simulation results show that the optimal policy outperforms other baseline policies when the environmental statistics are known. At the same time, the performance of the policy learned in the unknown environment is very close to the theoretical optimal policy. We also compare the age-reliable energy trade-off curves of the optimal updating policies under different energy supply conditions, which reflects the rationality of mixed energy supplies. The optimal policy can also be particularized to a special case, where the sensor can only utilize the harvested energy and the battery is unit-sized, and its performance coincides with the existing results in [23,25].

### 1.3. Organization

The rest of this paper is organized as follows. In Section 2, we introduce the model of the information update system and formulate the problem. In Section 3, we analyze the optimal policy when all the statistics are known. In Section 4, we aim to minimize the average cost of updating in an unknown environment. In Section 5, we present the simulation results. Finally, in Section 6, we conclude the paper.

## 2. System Model and Problem Formulation

### 2.1. System Model

**Figure 1:**
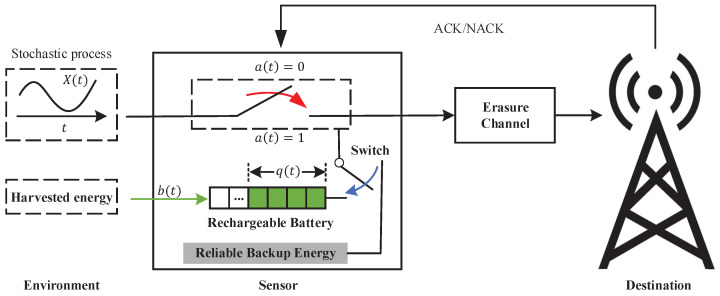
System model.

In this paper, we consider a point-to-point information update system, where a wireless sensor and a destination are connected by an erasure channel, as shown in Figure 1. The channel is assumed to be noisy and time invariant, and each update is corrupted with probability *p* during transmission (Note p∈(0,1)). Both the free harvested energy stored in the rechargeable battery and the reliable backup energy that needs to be paid can be used for real-time environmental status updates.

Without loss of generality, time is slotted with equal length and indexed by t=0,1,2⋯. At the beginning of each time slot, the sensor decides whether to generate and transmit an update to the destination or stay idle. The decision action at slot *t*, denoted by a[t], takes value from action set $A=\left\{0,1\right\}$, where a[t]=1 means that the sensor decides to generate and transmit an update to the destination while a[t]=0 means the sensor is idle. The destination will feed back an instantaneous ACK to the sensor through an error-free channel when it has successfully received an update and a NACK otherwise. We assume the above processes can be completed in one time slot. The destination keeps track of the environment status through the received updates. We apply the metric age of information to measure the freshness of the status information available at the destination.

#### 2.1.1. Age of Information

Age of Information (AoI) is defined as the elapsed time since the generation of the latest successfully received update [1,2,3]. Denote Δ[t] as the AoI of destination in time slot *t*. Then, we have

(1) Δ[t]=t−U[t].

where U[t] denotes the time slot when the most recently received update was generated before time slot *t*. In particular, the AoI will decrease to one if a new update is successfully received. Otherwise, it will increase by one. The evolution of AoI can be expressed as follows:

(2) $$\Delta \left[t+1\right]=\left\{\begin{array}{cc} 1, & \;\mathrm{successful}\;\mathrm{transmission},\\ \Delta [t]+1, & \;\mathrm{otherwise}. \end{array}\right.$$

**Figure 2:**
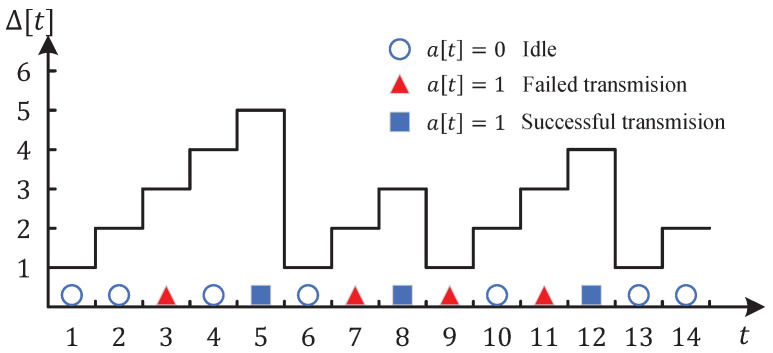
A sample path of AoI with initial age 1.

A sample path of AoI is depicted in Figure 2.

#### 2.1.2. Description of Energy Supply

We assume that only the sensor’s measurement and transmission process will consume energy and ignore other energy consumption. The energy unit is normalized, so the generation and transmission for each update will consume one energy unit. As previously described, the energy sources of the sensor include energy harvested from nature and reliable backup energy.

For the harvested energy, the sensor can store it in a rechargeable battery for later use. The maximum capacity of the rechargeable battery is *B* units (B>1). Considering the scarcity of energy in nature, the total energy harvested in one time slot may sometimes not reach an energy unit. So we consider using the Bernoulli process with the parameter λ to approximately capture the arrival process of harvested energy, which was also adopted in [41,42,43]. Let b[t] be the accumulated harvested energy in time slot *t*. That is, we have $\mathrm{Pr}\left\{b[t]=1\right\}=\lambda$ and $\mathrm{Pr}\left\{b[t]=0\right\}=1-\lambda$ in each time slot *t* (note λ∈(0,1)). Here, we assume that the energy arrival at each slot is independently and identically distributed. Time-correlated energy arrival processes, such as Markov process, will be considered in future work.

For reliable backup energy, we assume that it contains much more energy units than the rechargeable battery, so the energy it contains can be viewed as infinite. However, it needs to be used for a fee compared to the free renewable energy stored in the rechargeable battery. Therefore, when the stored renewable energy is not zero, the sensor will prioritize using it for status updates; otherwise, it will automatically switch to the reliable backup energy until the sensor has harvested energy. Defining the power of the rechargeable battery at the beginning of time slot *t* as the battery state q[t], then the evolution of battery state from time slot *t* to t+1 can be summarized as follows:

(3) q[t+1]=min{q[t]+b[t]−a[t]u(q[t]),B},

where u(·) is unit step function, which is defined as

(4) $$u\left(x\right)=\left\{\begin{array}{cc} 1, & \mathrm{if}\;x>0,\\ 0, & \mathrm{otherwise}. \end{array}\right.$$

Suppose that under reliable energy supply, the cost of generating and transmitting an update is a non-negative value $C_{r}$. Defining E[t] as the paid reliable energy cost at the time slot *t*, then we have

(5) $$E\left[t\right]=C_{r}a\left[t\right]\left(1-u\left(q\left[t\right]\right)\right).$$

### 2.2. Problem Formulation

Let Π denote the set of non-anticipative policies in which scheduling decision a[t] are made based on the action history ${\left\{a[k]\right\}}_{k=0}^{t-1}$, the evolution of AoI ${\left\{\Delta [k]\right\}}_{k=0}^{t}$, the evolution of battery state ${\left\{q[k]\right\}}_{k=0}^{t}$ as well as the system parameters (e.g., *p* and λ). In order to keep the information freshness at the destination, the sensor needs to send updates. However, due to the randomness of energy arrivals, the battery energy may sometimes be insufficient to support updates, and the sensor has to take energy from reliable backup energy. To balance the information freshness and the paid reliable backup energy cost, we aim to find the optimal information updating policy π∈Π that achieves the minimum of the time-average weighted sum of the AoI and the paid reliable backup energy cost. The problem is formulated as follows:

(6) $$\begin{array}{cc} \; & \min_{\pi \in \Pi }\underset{T\to \infty }{\lim\sup}\frac{1}{T}E_{\pi }\left\{\sum_{t=0}^{T-1}\left[\Delta [t]+\omega E\left[t\right]\right]\right\},\\ \; & s.t.\;(2),(3),(5), \end{array}$$

where ω is the pre-defined non-negative weighting factor. If ω = 0, the optimal policy is to update in each time slot, i.e., zero-wait policy [4]. Since the effect of energy can be ignored, if the rechargeable battery is not empty, the sensor uses the renewable energy; otherwise, the sensor will use the reliable energy directly. When ω>0, the optimal policy is non-trivial. So we will focus on the optimal policy for ω>0 in the rest of the paper. The smaller ω is, the more we attach importance to the system AoI; otherwise, the more emphasis is placed on the cost of reliable energy.

**Remark** **1.** *The optimal trade-off between age and reliable energy consumption can also be formulated as a constrained problem, where the reliable energy consumption serves as a constraint (not exceeding $E_{m}$) but not a penalty, and the goal is to minimize the long-term average age. By the Lagrangian method, it can be converted into an unconstrained weighted sum problem, where the Lagrangian multiplier is exactly the weight factor ω. So the solution proposed in this paper can be used. If there exists an ω such that the average reliable energy consumption in the minimum weighted sum is $E_{m}$, the optimal policy of the weighted sum problem also minimizes the long-term average age with the $E_{m}$ constraint. Otherwise, a randomized optimal policy for the constrained problem needs to be considered; see details in [44].*

## 3. Optimal Policy Analysis in A Known Environment

In this section, we aim to solve the problem (6) in a known environment and obtain the optimal policy. It is difficult to solve the original problem directly due to the random erasures and the temporal dependency in both AoI and battery state evolution. However, since the statistics such as channel erasure probability and EH probability are known, we can reformulate the original problem as a time-average cost MDP with infinite state space and analyze the structure of the optimal policy.

### 3.1. Markov Decision Process Formulation

According to the system description mentioned in the previous section, the MDP is formulated as follows:

- **State space**. The sensor’s state x[t] in slot *t* is a couple of the current destination AoI and the battery state, i.e., (Δ[t],q[t]). Define $B=\left\{0,1,\ldots ,B\right\}$. The state space $S=Z^{+}\times B$ is thus infinite countable.
- **Action space**. The sensor’s action a[t] in time slot *t* takes value from the action set $A=\left\{0,1\right\}$.
- **Transition probabilities**. Denote Pr(x[t+1]|x[t],a[t]) as the transition probability that current state x[t] transits to next state x[t+1] after taking action a[t]. Suppose the current state x[t]=(Δ,q) and action a[t]=a, then the transition probability is divided into two following cases conditioned on different values of action.
  ***Case 1***. a=0,
  (7) $$\left\{\begin{array}{cc} \mathrm{Pr}\{(\Delta +1,q+1)|(\Delta ,q),0\}=\lambda , & \;\mathrm{if}\;q<B,\\ \mathrm{Pr}\{(\Delta +1,B)|(\Delta ,B),0\}=1, & \;\mathrm{if}\;q=B,\\ \mathrm{Pr}\{(\Delta +1,q)|(\Delta ,q),0\}=1-\lambda , & \;\mathrm{if}\;q<B. \end{array}\right.$$
  ***Case 2***. a=1,
  (8) $$\left\{\begin{array}{cc} \mathrm{Pr}\{(\Delta +1,q)|(\Delta ,q),1\}=p\lambda , & \;\mathrm{if}\;q>0,\\ \mathrm{Pr}\{(1,q)|(\Delta ,q),1\}=(1-p)\lambda , & \;\mathrm{if}\;q>0,\\ \mathrm{Pr}\{\Delta +1,q-1)|(\Delta ,q),1\}=p(1-\lambda ), & \;\mathrm{if}\;q>0,\\ \mathrm{Pr}\{(1,q-1)|(\Delta ,q),1\}=(1-p)(1-\lambda ), & \;\mathrm{if}\;q>0,\\ \mathrm{Pr}\{(\Delta +1,1)|(\Delta ,0),1\}=p\lambda , & \;\mathrm{if}\;q=0,\\ \mathrm{Pr}\{(1,1)|(\Delta ,0),1\}=(1-p)\lambda , & \;\mathrm{if}\;q=0,\\ \mathrm{Pr}\{(\Delta +1,0)|(\Delta ,0),0\}=p(1-\lambda ), & \;\mathrm{if}\;q=0,\\ \mathrm{Pr}\{(1,0)|(\Delta ,0),0\}=(1-p)(1-\lambda ), & \;\mathrm{if}\;q=0. \end{array}\right.$$
  In both cases, the evolution of AoI still follows Equation (2) and the evolution of battery state follows Equation (3).
- **One-step cost**. For the current state x=(Δ,q), the one-step cost C(x,a) of taking action *a* is expressed by
  (9) $$C\left(x,a\right)=\Delta +\omega C_{r}a\left(1-u\left(q\right)\right).$$

After the above modeling, the original problem (6) is transformed into obtaining the optimal policy for the MDP to minimize the average cost in an infinite horizon:

(10) $$\min_{\pi \in \Pi }\underset{T\to \infty }{\lim\sup}\frac{1}{T}E_{\pi }\left\{\sum_{t=0}^{T-1}C\left(x\left[t\right],a\left[t\right]\right)\right\}.$$

Denote $\Pi_{SD}$ as the set of stationary deterministic policies. Given observation (Δ[t],q[t])=(Δ,q), the policy $\pi \in \Pi_{SD}$ selects action a[t]=π(Δ,q), where $\pi \left(\cdot \right):\left(\Delta ,q\right)\to \left\{0,1\right\}$ is a deterministic function from state space S to action space A. In the next section, we prove that there is an optimal stationary deterministic policy for the above unconstrained MDP with infinite countable state and action space.

### 3.2. The Existence of the Optimal Stationary Deterministic Policy

According to [15], we need to first address a discounted cost MDP, then relate it to the original average cost problem. Given an initial state $x\left[0\right]=\hat{x}$, the total expected discounted cost under a policy π is given by

(11) $$V_{\gamma }^{\pi }\left(\hat{x}\right)=\underset{T\to \infty }{\lim\sup}E_{\pi }\left\{\sum_{t=0}^{T-1}\gamma^{t}C\left(x\left[t\right],a\left[t\right]\right)|x\left[0\right]=\hat{x}\right\},$$

where the discounted factor is γ∈(0,1). Therefore, the problem of minimizing the expected discounted cost can be formulated as

(12) $$V_{\gamma }\left(\hat{x}\right)≜\min_{\pi \in \Pi }V_{\gamma }^{\pi }\left(\hat{x}\right),$$

where value function $V_{\gamma }\left(\hat{x}\right)$ denotes the minimum expected discounted cost. The policy is γ-optimal if it minimizes the above discounted cost. The optimality equation of $V_{\gamma }\left(\hat{x}\right)$ is introduced in Proposition 1.

**Proposition** **1.**

 *(a)*  *The optimal expected discounted cost $V_{\gamma }\left(\hat{x}\right)$ satisfies the Bellman equation as follows:*
(13) $$V_{\gamma }\left(\hat{x}\right)=\min_{a\in A}Q_{\gamma }\left(\hat{x},a\right),$$
  *where the state–action value function $Q_{\gamma }\left(\hat{x},a\right)$ is defined as*
(14) $$Q_{\gamma }\left(\hat{x},a\right)=C\left(\hat{x},a\right)+\gamma \sum_{x^{\prime }\in S}\mathrm{Pr}\left(x^{\prime }|\hat{x},a\right)V_{\gamma }\left(x^{\prime }\right).$$
 *(b)*  *The policy π determined by the right hand side of  (13) is γ-optimal, and $\pi \in \Pi_{SD}$*.
 *(c)*  *$V_{\gamma }\left(\hat{x}\right)$ can be solved by value iteration algorithm. Specifically, let $V_{\gamma ,n}\left(\hat{x}\right)$ be the cost-to-go function and $V_{\gamma ,0}\left(\hat{x}\right)=0$ for all state $\hat{x}\in S$. For all n≥1, we have:*
  (15) $$V_{\gamma ,n}\left(\hat{x}\right)=\min_{a\in A}Q_{\gamma ,n}\left(\hat{x},a\right),$$
  *where $Q_{\gamma ,n}\left(\hat{x},a\right)$ is obtained as follows:*
  (16) $$Q_{\gamma ,n}\left(\hat{x},a\right)=C\left(\hat{x},a\right)+\gamma \sum_{x^{\prime }\in S}\mathrm{Pr}\left(x^{\prime }|\hat{x},a\right)V_{\gamma ,n-1}\left(x^{\prime }\right).$$
  *Then the equation $\lim_{n\to \infty }V_{\gamma ,n}\left(\hat{x}\right)=V_{\gamma }\left(\hat{x}\right)$ holds for every state $\hat{x}$ and γ*.

**Proof.**  See Appendix A.    □

Now, we can show the monotonic properties of $V_{\gamma }\left(\hat{x}\right)$ in the following lemma by using (c) in Proposition 1.

**Lemma** **1.** *Given fixed channel erasure probability p and EH probability λ, then*

 *(a)*  *value function $V_{\gamma }\left(\Delta ,q\right)$ is **non-decreasing** in *Δ*, i.e., for any $1\leq \Delta_{1}\leq \Delta_{2}$ and any battery state q∈B, we have*
  (17) $$V_{\gamma }\left(\Delta_{1},q\right)\leq V_{\gamma }\left(\Delta_{2},q\right),$$
  *and*
  (18) $$V_{\gamma }\left(\Delta_{2},q\right)-V_{\gamma }\left(\Delta_{1},q\right)\geq \Delta_{2}-\Delta_{1}.$$
 *(b)*  *value function $V_{\gamma }\left(\Delta ,q\right)$ is **non-increasing** in q, i.e., for AoI Δ≥1 and any battery state $q\in \left\{0,1,\cdots ,B-1\right\}$, we have*
(19) $$V_{\gamma }\left(\Delta ,q\right)\geq V_{\gamma }\left(\Delta ,q+1\right),$$

**Proof.**  See Appendix B.    □

Based on the Proposition 1 and Lemma 1, we will verify the existence of the optimal stationary deterministic policy for the average cost problem (10) in the following theorem.

**Theorem** **1.** *There exists an optimal policy $\pi^{☆}\in \Pi_{SD}$ for the average cost MDP in (10). Moreover, for every state x, there exists a value function V(·):S→R and a unique constant $g^{☆}\in R$ such that:*

(20) $$g^{☆}+V\left(x\right)=\min_{a\in A}\left\{C\left(x,a\right)+\sum_{x^{\prime }\in S}\mathrm{Pr}\left(x^{\prime }|x,a\right)V\left(x^{\prime }\right)\right\},$$

*where $g^{☆}$ is the optimal average cost of problem (10) and satisfies $g^{☆}=\lim_{\gamma \to 1}\left(1-\gamma \right)V_{\gamma }\left(x\right)$ for every state x, and the value function V(x) satisfies*

(21) $$V\left(x\right)=\lim_{\gamma \to 1}\gamma V_{\gamma }\left(x\right)=\lim_{\gamma \to 1}V_{\gamma }\left(x\right)-g^{☆}=\underset{T\to \infty }{\lim\sup}\frac{1}{T}E_{\pi }\left\{\sum_{t=0}^{T-1}\left[C\left(x\left[t\right],a\left[t\right]\right)-g^{☆}\right]\right\}.$$

**Proof.**  See Appendix C.    □

Based on Theorem 1, we have the following corollary:

**Corollary** **1.** *The state–action value function Q(x,a) for the average cost is given as follows:*

(22) $$Q\left(x,a\right)=C\left(x,a\right)+\sum_{x^{\prime }\in S}\mathrm{Pr}\left(x^{\prime }|x,a\right)V\left(x^{\prime }\right),$$

*which is similar to $Q_{\gamma }\left(x,a\right)$ in (14) by letting γ→1. Then the optimal policy $\pi^{☆}\in \Pi_{SD}$ for the average cost MDP in (10) can be expressed as follows:*

(23) $$\pi^{☆}\left(x\right)=\arg\min_{a\in A}Q\left(x,a\right),\forall x\in S.$$

### 3.3. Structure Analysis of Optimal Policy

Before analyzing the structure of the optimal policy $\pi^{☆}$, we first prove some monotonic properties of the value function V(x) on different dimensions, which is summarized in the following lemma.

**Lemma** **2.** *Given fixed channel erasure probability p and EH probability λ, then*

 *(a)*  *value function V(Δ,q) is* ***non-decreasing*** *in Δ, i.e., for any $1\leq \Delta_{1}\leq \Delta_{2}$ and any battery state q∈B, we have*
(24) $$V\left(\Delta_{1},q\right)\leq V\left(\Delta_{2},q\right),$$
  *and*
(25) $$V\left(\Delta_{2},q\right)-V\left(\Delta_{1},q\right)\geq \Delta_{2}-\Delta_{1}.$$
 *(b)*  *value function V(Δ,q) is **non-increasing** in q, i.e., for AoI Δ≥1 and any battery state $q\in \left\{0,1,\cdots ,B-1\right\}$, we have*
(26) V(Δ,q)≥V(Δ,q+1),

**Proof.**  According to the (21), $V\left(x\right)=\lim_{\gamma \to 1}V_{\gamma }\left(x\right)-g$. Therefore, the monotonic properties of $V_{\gamma }\left(x\right)$ in Lemma 1 are also valid for V(x), which completes the proof.    □

Based on Lemma 2, we will derive the **proportional differential property** of the value function in Lemma 3.

**Lemma** **3.** *Given fixed channel erasure probability p and EH probability λ, then value function V(Δ,q) has the **proportional differential property**, i.e., the inequality*

(27) $$\frac{V(\Delta +1,q+1)-V(\Delta ,q+1)}{V(\Delta +1,q)-V(\Delta ,q)}\geq p$$

*holds for AoI Δ≥1 and any battery state $q\in \left\{0,1,\ldots ,B-1\right\}$.*

**Proof.**  See Appendix D.    □

With Corollary 1, Lemmas 2 and 3, we directly provide our main result in the following theorem.

**Theorem** **2.** *Assuming that the channel erasure probability p and EH probability λ are both fixed, there exists a threshold $\Delta_{q}\in Z^{+}$ for given battery state q, such that when $\Delta \;<\Delta_{q}$, the optimal action $\pi^{☆}\left(\Delta ,q\right)=0$, i.e., the sensor keeps idle; when $\Delta \geq \Delta_{q}$, the optimal action $\pi^{☆}\left(\Delta ,q\right)=1$, i.e., the sensor chooses to generate and transmit a new update.*

**Proof.**  See Appendix E.    □

Theorem 2 reveals the threshold structure of the optimal policy: if the optimal action in a certain state is to generate and transmit an update, then in the state with the same battery state and larger AoI, the optimal action must be the same. Note that the threshold $\Delta_{q}$ is actually determined by the channel erasure probability *p*, EH probability λ and pre-defined weighting factor ω. The closed-form expression of the threshold is difficult to be derived due to the complex transition probabilities. In the next section, we will show how to compute the optimal policy numerically.

### 3.4. Modified Relative Value Iteration Algorithm Design

In this section, we will propose a computationally efficient algorithm to find the optimal stationary deterministic policy based on the threshold structure.

Since the state space S is infinite, we will use a truncated space $S^{N}$ for approximation in practice, where $S^{N}=\left\{\left(\Delta ,q\right)|\Delta \leq N,\Delta \in Z^{+},q\in B\right\}$. It can be proved that when *N* is large enough, the optimal policy of the approximated MDP will be identical to that of the original problem [6].

However, the value iteration algorithm in Proposition 1 for the discounted cost problem cannot be applied to the average cost problem by letting γ=1. It does not converge because the value function V(·) in (20) is not unique. One can check if V(·) satisfies (20), a new function $V^{\prime }\left(\cdot \right)=V\left(\cdot \right)+c$ also satisfies (20), where c∈R. Therefore, we introduce a *relative value iterative* (RVI) algorithm to obtain the optimal policy of the approximate average cost MDP [45]. We choose a reference state $\hat{x}\in S^{N}$ and set $V_{0}\left(x\right)=0$ for all states $x\in S^{N}$ Then for all n≥0, we have

(28) $$V_{n+1}\left(x\right)=\min_{a\in A}Q_{n+1}\left(x,a\right),$$

and $Q_{n+1}\left(x,a\right)$ is obtained as follows:

(29) $$Q_{n+1}\left(x,a\right)=C\left(x,a\right)+\sum_{x^{\prime }\in S^{N}}\mathrm{Pr}\left(x^{\prime }|x,a\right)h_{n}\left(x^{\prime }\right),$$

where the differential value function is $h_{n}\left(x\right)=V_{n}\left(x\right)-V_{n}\left(\hat{x}\right)$. The equation $\lim_{n\to \infty }Q_{n}\left(x,a\right)=Q\left(x,a\right)$ holds for every state $x\in S^{N}$ and action a∈A. Finally, we compute the optimal policy by

(30) $$\pi^{☆}\left(x\right)=\arg\min_{a\in A}Q\left(x,a\right).$$

Note that the optimal policy is still of a threshold structure. The corresponding proof is similar to that of Theorem 2.

Moreover, based on the RVI algorithm, we can exploit this threshold structure to reduce the computational complexity. When the optimal policy of a state $x^{\prime }=\left(\Delta^{\prime },q^{\prime }\right)$ is 1, the optimal policy of state $x^{\prime }\in \left\{\left(\Delta ,q\right)|\Delta >\Delta^{\prime },\Delta \leq N,q=q^{\prime }\right\}$ will also be 1 without the need to calculate (30). Therefore, we propose a modified RVI algorithm, and the details are given in Algorithm 1.

**Algorithm 1** Modified relative value iteration algorithm.
**Input:**
  Iteration number *K*,  Iteration threshold ϵ,  Maximum of AoI *N*,  Maximum of battery state *B*,  Reference state $\hat{x}$.
**Output:**
  Optimal policy $\pi^{☆}\left(x\right)$ for all state x.1: **Initialization:**
$h_{0}\left(x\right)=0$, for all $x\in S^{N}$2: **for** episodes n=0,1,2,⋯,K **do**3:  **for** state $x\in S^{N}$ **do**4:   **for** action a∈A **do**5:    $Q_{n}\left(x,a\right)\leftarrow C\left(x,a\right)+\sum_{x^{\prime }\in S^{N}}\mathrm{Pr}\left(x^{\prime }|x,a\right)h_{n}\left(x^{\prime }\right)$// Update the state-action value function.6:    **end for**7:    $V_{n+1}\left(x\right)\leftarrow \min_{a\in A}Q_{n}\left(x,a\right)$// Update the value function.8:    $h_{n+1}\left(x\right)\leftarrow V_{n+1}\left(x\right)-V_{n+1}\left(\hat{x}\right)$// Update the differential value function.9:   **end for**10:   **if** $∥h_{n+1}\left(x\right)-h_{n}\left(x\right)∥\leq \epsilon ,\forall x\in S^{N}$ **then**11:    **for** $x=\left(\Delta ,q\right)\in S^{N}$ **do**12:     **if** $\pi^{☆}\left(\Delta -1,q\right)=1$ **then**13:     $\pi^{☆}\left(x\right)\leftarrow 1$, // Leverage the threshold structure of the optimal policy.14:     **else**15:      $\pi^{☆}\left(x\right)\leftarrow \arg\min_{a\in A}Q_{n}\left(x,a\right)$16:     **end if**17:    **end for**18:    **break**19:   **end if**20:  **end for**

## 4. Minimize Average Cost in an Unknown Environment

In the previous sections, we assumed that the channel erasure probability *p* and EH probability λ are known in advance. Thus, the *model-based* RVI method can be employed to obtain the optimal updating policy. However, statistics such as *p* and λ may be unknown and even time variant in many practical scenarios, which makes it impossible to apply modified RVI algorithm because the transition probabilities are not explicit and Equation (29) cannot be applied to estimate the state-action value function Q(x,a). In the field of reinforcement learning, alternatively, *model-free* methods can solve MDP problems with unknown transition probabilities. An example of a model-free algorithm is *Q-learning* [46]. *Q*-learning finds an optimal policy in the sense of maximizing the expected value of the total reward over any and all successive steps. However, it is only designed for discounted MDP. For the average cost problem in (10), we employ an average cost *Q*-learning algorithm. The basic idea of this algorithm comes from the *SMART* algorithm in [47], which is a model-free reinforcement learning algorithm proposed for semi-Markov decision problems (SMDP) under the average-reward criterion. We modify it to fit the average cost MDP problem.

The state–action value function Q(x,a) is essential for solving the optimal policy. When the model is unknown, as long as Q(x,a) can be estimated accurately, the optimal policy can also be obtained immediately by (30). So the key question is how to estimate the Q(x,a) function, or equivalently, the value of all state–action pairs. Similar to *Q*-learning, the average cost *Q*-learning algorithm uses the minimum value of the next state–action pairs to update the value of the current state–action pair. Moreover, it needs to estimate the shift value *g* by averaging all immediate cost.

Specifically, the average cost *Q*-learning algorithm learns Q(x,a) by episodes. Each episode contains several iterations, and each iteration corresponds to one time slot. Then in the *n*th time slot of an episode, the algorithm first observes the current state x[n]=(Δ[n],q[n]), selects an action a[n] according to the ϵ-greedy policy:

(31) $$a\left[n\right]=\left\{\begin{array}{cc} \arg\min_{a\in A}Q\left(x\left[n\right],a\right), & \mathrm{with}\;\mathrm{probability}\;1-\epsilon ,\\ \mathrm{random}\;\mathrm{action}, & \mathrm{otherwise}. \end{array}\right.$$

By (9), the immediate cost $C\left[n\right]=\Delta \left[n\right]+\omega C_{r}a\left[n\right]\left(1-u\left(q\left[n\right]\right)\right)$ occurs, and the system will transit to the next state x[n+1]. The value of Q(x[n],a[n]) is updated as follows:

(32) $$Q\left(x\left[n\right],a\left[n\right]\right)=\left(1-\alpha \left[n\right]\right)Q\left(x\left[n\right],a\left[n\right]\right)+\alpha \left[n\right](C\left[n\right]-g+\min_{a\in A}Q\left(x\left[n+1\right],a\right)),$$

where α[n] is the learning rate. The shift value *g* is updated as follows:

(33) g=(1−β[n])g+β[n]C[n]

where $\beta \left[n\right]=\frac{1}{n}$. The details are given in Algorithm 2. We leverage the parameter ϵ to balance exploration and exploitation. As the number of epochs increases, the learned Q(x,a) value will approach its true value, so we can gradually decrease ϵ to 0 to reduce invalid exploration. At the same time, the shift value *g* will also be close to the optimal average cost $g^{☆}$ in (20). Note that in [47], the shift value *g* is updated only in a non-exploratory time slot. Here we update it by simply averaging all cost, similar to [48]. The performance comparison of the average cost *Q*-learing algorithm and modified RVI algorithm is shown in the next section.

**Algorithm 2** Average cost *Q*-learning algorithm.
**Input:**
 Maximum number of episodes *K*, Maximum iteration number of an episode $N_{e}$, Maximum of AoI *N*, Maximum of battery state *B*, Initial value of $Q^{N\times B\times 2}\leftarrow 0$, Initial value of ϵ←0, Initial value of the shift value g←0.
**Output:**
 Learned policy π(x) for all state x, Average cost $g^{☆}$ by following the policy π.1: **for** episodes k=0,1,2,⋯,K **do**2:  g←0 // Initialize the shift value at the beginning of every episode.3:  **for** $n=1,2,\cdots ,N_{e}$ **do**4:   Observe the current state x[n]5:   Select an action a[n] according to ϵ-greedy policy in (31)6:   Calculate immediate cost $C\left[n\right]\leftarrow \Delta \left[n\right]+\omega C_{r}a\left[n\right]\left(1-u\left(q\left[n\right]\right)\right)$7:   Observe the next state x[n+1]8:   $\alpha \left[n\right]\leftarrow \frac{1}{\sqrt{n}}$9:   $Q\left(x\left[n\right],a\left[n\right]\right)\leftarrow \left(1-\alpha \left[n\right]\right)Q\left(x\left[n\right],a\left[n\right]\right)+\alpha \left[n\right](C\left[n\right]-g+\min_{a\in A}Q\left(x\left[n+1\right],a\right))$ // Update the state-action value function.10:   $\beta \left[n\right]\leftarrow \frac{1}{n}$11:   g←(1−β[n])g+β[n]C[n] // Update the shift value.12:  **end for**13:  Decrease ϵ14: **end for**15: **for**
$x=\left(\Delta ,q\right)\in S^{N}$
**do**16:  $\pi \left(x\right)\leftarrow \arg\min_{a\in A}Q\left(x,a\right)$// Calculate the learned policy π.17: **end for**

## 5. Numerical Results

In this section, we first show the threshold structure of the optimal policy by the simulation results. Then we compare the performance of the optimal policy with the following representative policies under different system parameters:

- Zero-wait policy [4]. The sensor generates and transmits an update in every time slot.
- Periodic policy. The sensor periodically generates and sends updates to the destination.
- Randomized policy. The sensor chooses to send an update or stay idle in each time slot with the same probability.
- Energy first policy. The sensor only uses the harvested energy, that is, as long as the battery state is not zero, it will choose to sense and send updates, otherwise it will remain idle.

Moreover, we will show the average cost *Q*-learning algorithm performs very close to the modified RVI with known statistics. We will also compare age and reliable energy cost trade-off curves of the optimal updating policies under EH supply, reliable energy supply and mixed energy supplies. Finally, we compare the performance of the optimal policy under the only EH supply and unit-sized battery setting with the prior results in [23,25].

### 5.1. Simulation Setup

In our simulations, we set the maximum of AoI N=500, and the maximum of battery state B=20. So the finite state space $S^{N}=\left\{\left(\Delta ,q\right)|\Delta \leq 500,\Delta \in Z^{+},q\in B\right\}$. The cost of reliable energy $C_{r}$ for one update is equal to 2. For the modified RVI algorithm, we set the iteration number K=1000, iteration threshold $\epsilon =10^{-5}$ and reference state $\hat{x}=\left(1,B\right)$. The optimal policy and other baseline policies are run for T= 10,000 time slots to compute the average cost. For the average cost *Q*-learning algorithm, we set the total number of episodes K=1000, and the maximum iteration number in an episode $N_{e}=$ 100,000.

### 5.2. Results

**Figure 3:**
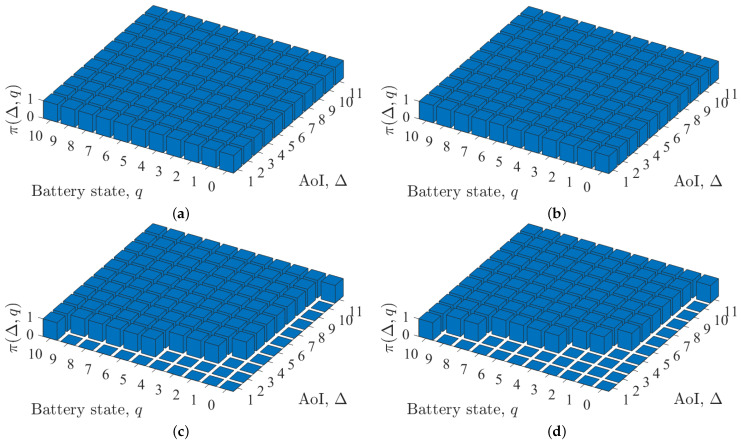
Optimal policy conditioned on different parameters: (**a**) ω=0.1, p=0.2, λ=0.5, (**b**) ω=0.1, p=0.4, λ=0.5, (**c**) ω=10, p=0.2, λ=0.5 and (**d**) ω=10, p=0.4, λ=0.5.

Figure 3 shows the optimal policy under different system parameters. All the subfigures in Figure 3 exhibit the threshold structure described in Theorem 2. Intuitively, when ω is very small, the optimal action for every state should be 1, and when ω is very large, the optimal action for battery state q=0 should be 0. It can be observed from Figure 3a,b that when ω is small (i.e., ω=0.1), the optimal policy is to update for every state, which is exactly the zero-wait policy. Figure 3 also shows that when ω is relatively large (e.g., ω=10), and the AoI is small, even if the battery state is not zero, the optimal action in the corresponding state is to keep idle. When the AoI is large or the battery state is large, the optimal action is to measure and send updates. Moreover, in all the subfigures, the threshold $\Delta_{q}$ keeps monotonically non-increasing with the battery state *q*. However, this conclusion has not been rigorously proven.

**Figure 4:**
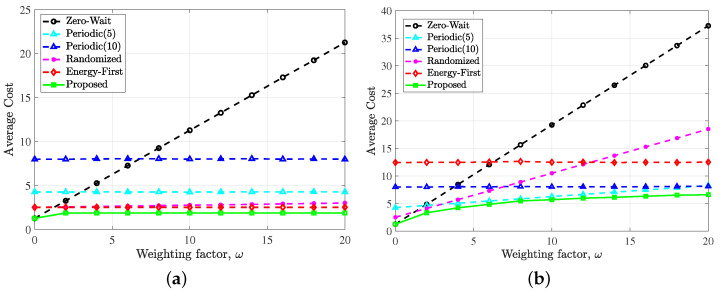
Performance comparison of the proposed optimal policy, zero-wait policy, periodic policy (period = 5), periodic policy (period = 10), randomized policy and energy first policy versus the weighting factor ω with simulation conditions: (**a**) p=0.2, λ=0.5 and (**b**) p=0.2, λ=0.1.

Figure 4 shows the time average cost with respect to ω under different policies. Here, we set the period of the periodic policy to 5 and 10 for comparison without loss of generality. It can be found that under different weighting factor ω, the optimal policy proposed in this paper can obtain the minimum long-term average cost compared with the other policies, which indicates the best trade-off between the average AoI and the cost of reliable energy. When ω tends to 0, the zero-wait policy tends to be optimal. Since there is no need to consider the update cost brought by paid reliable backup energy, the optimal policy should maximize the utilization of the updating opportunities.

It can also be observed from Figure 4 that the growth of the optimal policy curve slows down as ω increases. This is because the optimal policy in the case of large ω does not tend to use the reliable energy when battery state q=0, but prefers to wait for harvested energy, as shown in Figure 3. Since the EH probability is constant, the average AoI does not change much, resulting in no significant increase in the total average cost. Comparing Figure 4a,b, it is found that the larger the λ, the smaller the average cost variation with ω. This is because there is not much opportunity for the sensor to use reliable energy in the case of sufficient harvested energy.

**Figure 5:**
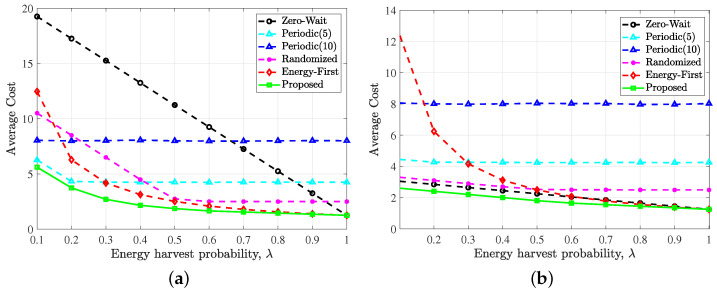
Performance comparison of the proposed optimal policy, zero-wait policy, periodic policy (period = 5), periodic policy (period = 10), randomized policy and energy first policy versus the EH probability λ with simulation conditions: (**a**) p=0.2, ω=10 and (**b**) p=0.2, ω=1.

Figure 5 reveals the impact of EH probabilities λ. In Figure 5a,b, we set p=0.2, ω=10 and p=0.2,ω=1, respectively.

It can also be found from both Figure 5a,b that the proposed optimal update policy outperforms all other policies under different EH probability. The interesting point is that when the EH probability tends to 1, i.e., energy arrives in each time slot, the performance of the zero-wait policy and the energy first policy is equal to the optimal policy, while there is still a performance gap between the optimal policy and the other two polices. This is intuitive because when the free harvested energy is sufficient, the optimal policy must be to generate and transmit updates in every time slot. However, the periodic policy and the randomized policy still keep idle in many time slots, which will lead to a higher average AoI and thus increase the average cost. Results show that the performance of zero-wait policy approaches the optimal policy for large λ, which is consistent with our findings in Figure 4.

**Figure 6:**
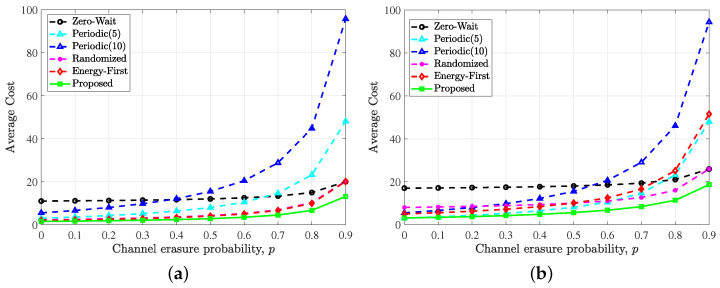
Performance comparison of the proposed optimal policy, zero-wait policy, periodic policy (period = 5), periodic policy (period = 10), randomized policy and energy first policy versus the erasure probability *p* with simulation conditions: (**a**) λ=0.5, ω=10 and (**b**) λ=0.2, ω=10.

In Figure 6, we compare the five policies under different channel erasure probability *p*.

It can be found that when the erasure probability increases from 0 to 0.9, the proposed optimal update policy always performs better than the other baseline policies. As *p* tends to 1, the average cost under all policies theoretically tends to infinity because all updates will be erased by the noisy channel and cannot be received by the destination. The simulation results confirmed this conjecture. Comparing Figure 6a,b, we can observe that when λ is large, the energy-first strategy will be close to the optimal strategy, which is also illustrated in Figure 5.

**Figure 7:**
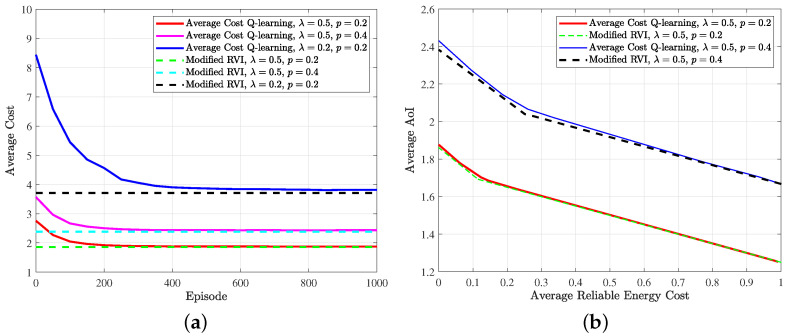
(**a**) Performance of the average cost *Q*-learning with respect to the modified RVI algorithm under different system parameters (ω=10); (**b**) age–energy trade-off curves computed by the average cost *Q*-learning and modified RVI algorithm.

Figure 7 shows the performance of the average cost *Q*-learning algorithm. In every episode, the shift value *g* of the last inner iteration is recorded as the average cost. It can be found from Figure 7a that the average cost achieved by Algorithm 2 converges to that obtained by the modified RVI algorithm under different EH probability λ and channel erasure probability *p*. The age–energy trade-off is shown in Figure 7b. By fixing λ and *p* and changing ω from 0 to 1000, we run the modified RVI algorithm and average cost *Q*-learning algorithm to obtain the corresponding trade-off curve. It can be found that the curve obtained by the average cost *Q*-learning algorithm is very close to the optimal trade-off curve under the same condition, which further verifies the near-optimal performance of the average cost *Q*-learning algorithm in an unknown environment.

**Figure 8:**
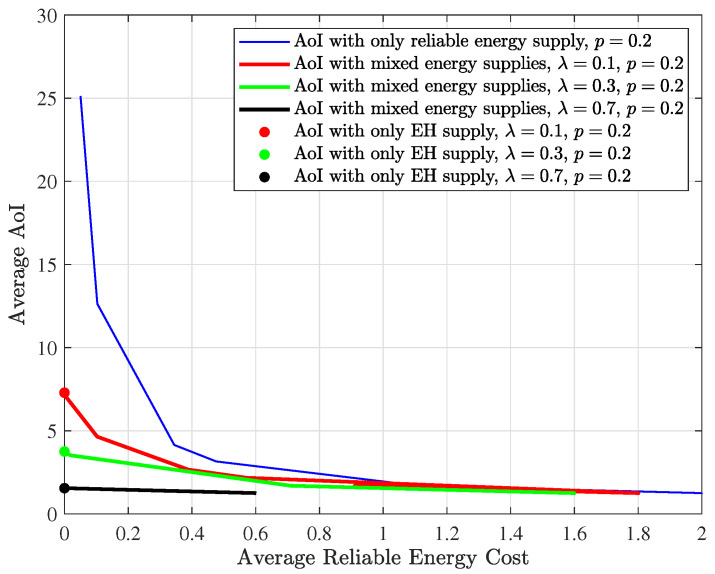
Age-reliable energy trade-off for different energy supplies: mixed energy supply, reliable energy supply and EH supply. The channel erasure probability p=0.2, and the EH probability λ is set as 0.1, 0.3 and 0.7, respectively.

Figure 8 shows the optimal age and reliable energy cost trade-off curves for different energy supplies. By fixing EH probability λ and channel erasure probability *p* and changing ω from 0 to 10,000, we run the modified RVI algorithm to get the optimal trade-off curve for mixed energy supplies. By letting EH probability λ=0 and following the same steps, we can obtain the optimal trade-off curve for reliable energy supply. By letting weighting factor ω go to infinity, we can theoretically obtain the optimal trade-off “curve” corresponding to the EH supply. The “curve” contains only one point because the reliable energy consumption can only be 0 for the EH supply case. It should be noted that ω cannot be infinite in a simulation. Instead, we can set ω to a relatively large number (e.g., 10,000). To facilitate comparison, the channel erasure probability is set as p=0.2, and the EH probability λ is set as 0.1, 0.3 and 0.7. It can be observed that the curves for the mixed energy supplies are always at the lower left of the curve for relying solely on reliable energy, which indicates that under the same average AoI, the reliable energy required by the system under the mixed energy supplies is smaller, and under the same reliable energy consumption, the AoI of the system under the mixed energy supplies is lower. The mixed energy design also achieves lower AoI than that with only EH, at the cost of paying for reliable energy. The optimal updating policy proposed in this paper makes full use of the harvested energy.

**Figure 9:**
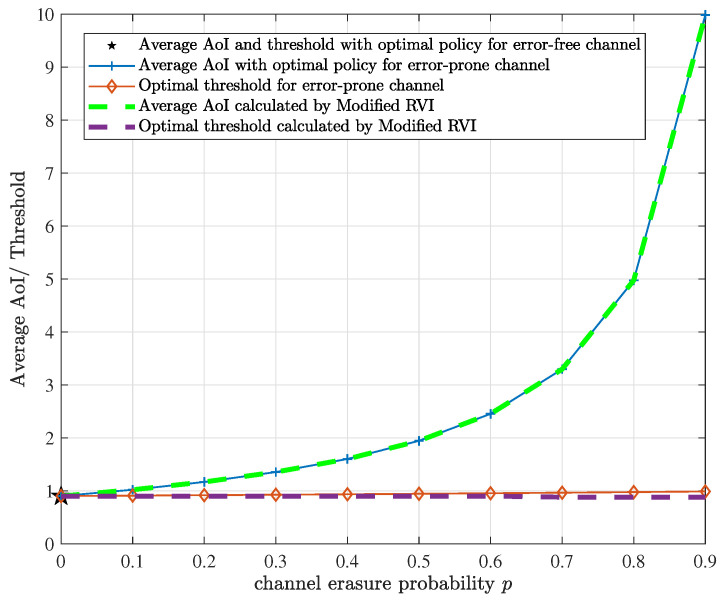
AoI and threshold with the proposed optimal policy for a special case where the sensor only uses the harvested energy and the battery capacity B=1, and those with a unit-sized battery in [23,25] (error-free channel case and error-prone channel case, respectively), vs. the channel erasure probability *p*.

Figure 9 compares the performance of the optimal policy with the prior results in [23,25] for a special case where the sensor only uses the harvested energy and the battery capacity B=1. Both [23,25] considered a continuous-time model, i.e., the energy arrival process is a Poisson process with an arrival rate of Λ energy units per *time unit* (TU), and proved that the optimal policies are threshold structure, in which a new update is generated and transmitted only if the time until the next energy arrival since the latest successful transmission exceeds a certain threshold. Specifically, [23] (Theorem 4, Equation (13)) provided the average AoI and threshold in closed-form under the optimal update policy for any energy arrival rate Λ in the error-free channel case. It is interesting that the optimal average AoI and the corresponding threshold are equal. Ref. [25] (Theorem 4, Equation (14)) extended the results of [23] to an error-prone channel case, while the energy arrival rate Λ is assumed to be 1. So we first show the results of [23,25] vs. different channel erasure probability *p* in Figure 9, where the energy arrival rate Λ=1. It should be emphasized that the unit of the average AoI and threshold is TU. According to Theorems 1 and 2 in this paper, the optimal update policy exists and admits a threshold structure for any EH probability λ, channel erasure probability *p*, weighting factor ω and battery capacity *B*. This conclusion is based on the discrete-time model, i.e., the energy arrives as a Bernoulli process with parameter λ, which is different from the continuous-time model in [23,25], and the reliable backup energy is also considered. However, by the choice of some parameters (large ω, small λ), our results can be a good approximation of the results in [23,25]. First, by choosing a large ω, the reliable energy will almost never be used, and equivalently, only the EH supply exists. Secondly, by choosing a small λ, the Poisson process can be approximated as a Bernoulli process. This is because for a Poisson process with parameter Λ, we can discretize a TU into *n* small time slots of equal length, then according to probability theory, when *n* is large enough, the energy arrival process within a time slot can be approximated as a Bernoulli process with parameter λ=Λ/n, which is relatively small. In our simulation, we set the battery capacity B=1, and take λ=0.1 (i.e., n=10) and ω= 10,000. By changing the channel erasure probability *p*, we can run the modified RVI algorithm to compute the minimum average AoI and the optimal threshold. It needs to be mentioned that the unit of them is a time slot. For comparison, we need to divide their values by *n* to obtain the average AoI and threshold in TU. The final results are shown by the dashed lines in Figure 9. It can be observed that the results of this paper are extremely close to the explicit results in [23,25], which verifies the correctness of the analysis and also reflects the generality of our system model.

## 6. Conclusions

In this paper, we studied the optimal updating policy for an information update system, where a wireless sensor sends updates over an erasure channel using both harvested energy and reliable backup energy. Theoretical analysis indicates the threshold structure of the optimal policy and simulation results verify its performance. For the practical case where the statistics, such as the EH probability and channel erasure probability, are unknown in advance, a learning-based algorithm is proposed to compute the updating policy. Simulation results show its performance is close to that of the optimal policy. With the optimal policy, the design of mixed energy supplies can make full use of harvested energy and achieve the best age–energy trade-off. In future work, we will focus on the timeliness of the multi-sensor system under mixed energy supplies.

## Figures and Tables

**Table 1:** Comparative summary of the most related works in contrast to our paper.

	Ref	[23]	[25]	Our
Feature				
Energy supply		EH	EH	EH + reliable energy
Battery capacity		Finite-sized	Unit-sized	Finite-sized
Wireless channel		Error-free	Error-prone	Error-prone
Optimization objective		AoI	AoI	AoI-reliable energy trade-off

**Figure A1:**
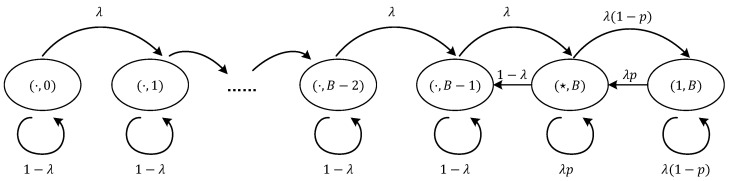
A simplified Markov chain of system states under the lazy policy. Note that (1,B) is the reference state. (·,1) means the state set $\left\{\left(\Delta ,q\right)|\Delta \in Z^{+},q=1\right\}$, (☆,B) means the state set $\left\{\left(\Delta ,q\right)|\Delta \in Z^{+},\Delta >1,q=B\right\}$ and so on for the rest.

## Data Availability

Not applicable.

## Abbreviations

The following abbreviations are used in this manuscript:

AoI	Age of Information
EH	Energy Harvesting
MDP	Markov Decision Process
RVI	Relative Value Iteration
SMART	Semi-Markov Average Reward Technique
SMDP	Semi-Markov Decision problems

## Appendix A. Proof of Proposition 1

According to [15], the proof of Proposition 1 is equivalent to proving that there is a stationary deterministic policy π such that the expected discounted cost $V_{\gamma }^{\pi }\left(x\right)$ is finite for all x, γ. So we can select a policy π which chooses to keep idle in each time slot. Then by (11), for any state x=(Δ,q)∈S and γ∈(0,1), we have

(A1) $$\begin{array}{cc} V_{\gamma }^{\pi }\left(x\right) & =E_{\pi }\left\{\sum_{t=0}^{\infty }\gamma^{t}C\left(x\left[t\right],a\left[t\right]\right)|x\left[0\right]=x\right\}\\ \; & =\sum_{t=0}^{\infty }\gamma^{t}C\left(x\left[t\right],a\left[t\right]\right)\\ \; & =\sum_{t=0}^{\infty }\gamma^{t}\left(\Delta +t\right)\\ \; & =\frac{1}{1-\gamma }\left(\Delta +\frac{\gamma }{1-\gamma }\right)<\infty , \end{array}$$

which completes the proof.

## Appendix B. Proof of Lemma 1

The proof requires the use of value iteration algorithm(VIA) and mathematical induction. According to (c) in Proposition 1, The specific iteration process of VIA is as follows:

(A2) $$\left\{\begin{array}{c} V_{\gamma ,0}\left(x\right)=0,\\ Q_{\gamma ,k}\left(x,a\right)=C\left(x,a\right)+\gamma \sum_{x^{\prime }\in S}\mathrm{Pr}\left(x^{\prime }|x,a\right)V_{\gamma ,k}\left(x^{\prime }\right),\\ V_{\gamma ,k+1}\left(x\right)=\min_{a\in A}Q_{\gamma ,k}\left(x,a\right), \end{array}\right.$$

where $k\in Z^{+}$. For any state x∈S, $V_{\gamma ,k}\left(x\right)$ will converge when *k* goes into infinity:

(A3) $$\lim_{k\to \infty }V_{\gamma ,k}\left(x\right)=V_{\gamma }\left(x\right).$$

Then we will use mathematical induction to prove the monotonicity of the value function in each component.

First let us tackle part (a) of Lemma 1.

For (17), we can verify that the inequality $V_{\gamma ,1}\left(\Delta_{1},q\right)\leq V_{\gamma ,1}\left(\Delta_{2},q\right)$ holds when k=1. Then we assume that at the *k*th step of the induction method, the following formula holds:

(A4) $$V_{\gamma ,k}\left(\Delta_{1},q\right)\leq V_{\gamma ,k}\left(\Delta_{2},q\right),\forall \Delta_{1}\leq \Delta_{2}.$$

So the next formula that needs to be verified is

(A5) $$V_{\gamma ,k+1}\left(\Delta_{1},q\right)\leq V_{\gamma ,k+1}\left(\Delta_{2},q\right),\forall \Delta_{1}\leq \Delta_{2}$$

Since $V_{\gamma ,k+1}\left(x\right)=\min_{a\in A}Q_{\gamma ,k}\left(x,a\right)$, we need to bring out $Q_{\gamma ,k}\left(x,a\right)$ first. Due to the complexity of the transition probabilities and one-step cost function, we need to discuss the following three cases: q=0, 0<q<B and q=B. For the sake of brevity, we only give the calculation details of the case 0<q<B, and the other two cases can be verified by following the exact same steps.

According to transition probability (7) and (8), we have the state-action value function $Q_{\gamma ,k}\left(\Delta ,q,0\right)$ and $Q_{\gamma ,k}\left(\Delta ,q,1\right)$ as follows:

(A6) $$\begin{array}{cc} Q_{\gamma ,k}\left(\Delta ,q,0\right)=\Delta & +\gamma \lambda V_{\gamma ,k}\left(\Delta +1,q+1\right)+\gamma \left(1-\lambda \right)V_{\gamma ,k}\left(\Delta +1,q\right), \end{array}$$

and

(A7) $$\begin{array}{cc} Q_{\gamma ,k}\left(\Delta ,q,1\right)=\Delta & +\gamma p\lambda V_{\gamma ,k}\left(\Delta +1,q\right)+\gamma p\left(1-\lambda \right)V_{\gamma ,k}\left(\Delta +1,q-1\right)\\ \; & +\gamma \left(1-p\right)\lambda V_{\gamma ,k}\left(1,q\right)+\gamma \left(1-p\right)\left(1-\lambda \right)V_{\gamma ,k}\left(1,q-1\right). \end{array}$$

Because $V_{\gamma ,k}\left(\Delta ,q\right)$ is assumed to be non-decreasing function with respect to Δ for any fixed *q*, it is obvious that both $Q_{\gamma ,k}\left(\Delta ,q,0\right)$ and $Q_{\gamma ,k}\left(\Delta ,q,1\right)$ are non-decreasing with respect to Δ. Therefore, for any $\Delta_{1}\leq \Delta_{2}$, we have

(A8) $$\begin{array}{cc} V_{\gamma ,k+1}\left(\Delta_{1},q\right) & =\min_{a\in A}\left\{Q_{\gamma ,k}\left(\Delta_{1},q,a\right)\right\}\\ \; & =\min\left\{Q_{\gamma ,k}\left(\Delta_{1},q,0\right),Q_{\gamma ,k}\left(\Delta_{1},q,1\right)\right\}\\ \; & \leq \min\left\{Q_{\gamma ,k}\left(\Delta_{2},q,0\right),Q_{\gamma ,k}\left(\Delta_{2},q,1\right)\right\}\\ \; & =V_{\gamma ,k+1}\left(\Delta_{2},q\right). \end{array}$$

As a result, with the induction we prove that $V_{\gamma ,k}\left(\Delta ,q\right)$ is a non-decreasing function with respect to Δ for any $q\in \left\{1,\cdots ,B-1\right\}$, i.e., the Equation (A4) holds. When *k* goes to infinity, combining (A3) and (A4), we prove that (17) holds in the case 0<q<B. In the other two cases, (17) still holds. So we have proved that (17) holds for any q∈B.

For (18), it is easy to yield

(A9) $$\begin{array}{cc} Q_{\gamma }\left(\Delta_{2},q,0\right) & -Q_{\gamma }\left(\Delta_{1},q,0\right)=\Delta_{2}-\Delta_{1}\\ \; & +\gamma \lambda [V_{\gamma }\left(\Delta_{2}+1,q+1\right)-V_{\gamma }\left(\Delta_{1}+1,q+1\right)]\\ \; & +\gamma \left(1-\lambda \right)[V_{\gamma }\left(\Delta_{2}+1,q\right)-V_{\gamma }\left(\Delta_{1}+1,q\right)]\\ \overset{\left(a\right)}{\geq } & \Delta_{2}-\Delta_{1}, \end{array}$$

and

(A10) $$\begin{array}{cc} Q_{\gamma }(\Delta_{2}, & q,1)-Q_{\gamma }\left(\Delta_{1},q,1\right)=\Delta_{2}-\Delta_{1}\\ \; & +\gamma p\lambda [V_{\gamma }\left(\Delta_{2}+1,q\right)-V_{\gamma }\left(\Delta_{1}+1,q\right)]\\ \; & +\gamma p\left(1-\lambda \right)[V_{\gamma }\left(\Delta_{2}+1,q-1\right)-V_{\gamma }\left(\Delta_{1}+1,q-1\right)]\\ \; & +\gamma \left(1-p\right)\lambda [V_{\gamma }\left(1,q\right)-V_{\gamma }\left(1,q\right)]\\ \; & +\gamma \left(1-p\right)\left(1-\lambda \right)[V_{\gamma }\left(1,q-1\right)-V_{\gamma }\left(1,q-1\right)]\\ \overset{\left(b\right)}{\geq } & \Delta_{2}-\Delta_{1}, \end{array}$$

where (a) and (b) are due to (17). Since $V_{\gamma }\left(x\right)=\min_{a\in A}Q_{\gamma }\left(x,a\right)$, we prove that Equation (18) holds for all $q\in \left\{1,\cdots ,B-1\right\}$. Through the same proof process, it can also be verified that (18) is also valid when q=0 and q=B. Therefore, we have completed the proof of part (a).

Second, we will tackle the part (b) of Lemma 1.

For (19), according to the exact same mathematical induction we have applied to (17), we can also verify that Equation (19) holds. Due to limited space, the details are omitted here.

Hence, we have completed the whole proof.

## Appendix C. Proof of Theorem 1

By Proposition 4 in [15], it suffices to show that the following four conditions hold:

(1): For every state x and discount factor γ, the discount value function $V_{\gamma }\left(x\right)$ is finite.
(2): There exists a non-negative value *L* such that $L\leq h_{\gamma }\left(x\right)$ for all x and γ, where $h_{\gamma }\left(x\right)=V_{\gamma }\left(x\right)-V_{\gamma }\left(\hat{x}\right)$, and $\hat{x}$ is a reference state.
(3): There exists a non-negative value M(x), such that $h_{\gamma }\left(x\right)\leq M\left(x\right)$ for every x and γ.
(4): The inequality $\sum_{x^{\prime }\in S}Pr\left(x^{\prime }|x,a\right)M\left(x^{\prime }\right)<\infty$ holds for all x and *a*.

For condition (1), recall that we have verified that there exists a stationary deterministic policy π such that the expected discounted cost $V_{\gamma }^{\pi }$ is finite in the proof of Proposition 1, and here we will extend this conclusion to any policy π∈Π. For any non-anticipative policy π and state x=(Δ,q), we have

(A11) $$C\left(x\left[t\right],a\left[t\right]\right)=\Delta +\omega C_{r}a\left(1-u\left(t\right)\right)\leq \Delta +\omega C_{r}.$$

Since the AoI grows linearly at most, for any state x=(Δ,q) and discounted factor γ, we have

(A12) $$\begin{array}{cc} V_{\gamma }\left(x\right) & =\min_{\pi \in \Pi }V_{\gamma }^{\pi }\left(x\right)=\min_{\pi \in \Pi }E_{\pi }\left\{\sum_{t=0}^{\infty }\gamma^{t}C\left(x\left[t\right],a\left[t\right]\right)|x\left[0\right]=\left(\Delta ,q\right)\right\}\\ \; & \leq \sum_{t=0}^{\infty }\gamma^{t}\left(\Delta +t+\omega C_{r}\right)\\ \; & =\frac{1}{1-\gamma }\left(\Delta +\omega C_{r}+\frac{\gamma }{1-\gamma }\right)<\infty , \end{array}$$

which verifies condition (1).

Next let us focus on condition (2). By (17) and (19) in Lemma 1, $V_{\gamma }\left(\Delta ,q\right)$ is non-decreasing with regard to age Δ and non-increasing with regard to battery state *q*. Hence, we can choose L=0 and reference state $\hat{x}=\left(1,B\right)$. Then we have $L=0\leq V_{\gamma }\left(x\right)-V_{\gamma }\left(\hat{x}\right)=h_{\gamma }\left(x\right)$, which verifies condition (2).

To prove that condition (3) holds, we need to introduce the following lemma:

**Lemma** **A1.**
*Denote $\hat{x}=\left(1,B\right)$ as the reference state, and $T=\underset{}{\inf}\left\{t:t\geq 0,x\left[t\right]=\hat{x}\right\}$ as the first hitting time from the initial state x to $\hat{x}$. Under the following lazy policy $\pi^{\prime }$:*

(A13)
$$\pi^{\prime }\left(\Delta ,q\right)=\left\{\begin{array}{cc} 1, & \mathrm{if}\;q=B,\\ 0, & \mathrm{otherwise}, \end{array}\right.$$

*the expected discounted cost from x to $\hat{x}$ is finite for all initial state x∈S, i.e.,*

(A14)
$$C^{\pi^{\prime }}\left(x\right)=E_{\pi^{\prime }}\left\{\sum_{t=0}^{T-1}\gamma^{t}C\left(x\left[t\right],a\left[t\right]\right)|x\left[0\right]=x\right\}<\infty .$$

*Note that if $x=\hat{x}$, $C^{\pi^{\prime }}\left(x\right)=0$.*

**Proof.** see Appendix F. □

Considering a mixed non-anticipative policy $\pi^{m}$ consisting of $\pi^{\prime }$ and optimal policy $\pi^{☆}$ for (12) from the initial state x as follows,

(A15) $$\pi^{m}\left(x\left[t\right]\right)=\left\{\begin{array}{cc} \pi^{\prime }\left(x\left[t\right]\right), & \mathrm{if}\;t<T,\\ \pi^{☆}\left(x\left[t\right]\right), & \mathrm{otherwise}, \end{array}\right.$$

we have

(A16) $$\begin{array}{cc} V_{\gamma }\left(x\right) & \leq V_{\gamma }^{\pi^{m}}\left(x\right)=E_{\pi^{\prime }}\left\{\sum_{t=0}^{T-1}\gamma^{t}C\left(x\left[t\right],a\left[t\right]\right)|x\left[0\right]=x\right\}+E_{\pi^{☆}}\left\{\sum_{t=T}^{\infty }\gamma^{t}C\left(x\left[t\right],a\left[t\right]\right)|x\left[T\right]=\hat{x}\right\}\\ \; & \overset{\left(a\right)}{=}C^{\pi^{\prime }}\left(x\right)+E_{\pi^{☆}}\left\{\gamma^{T}V_{\gamma }\left(\hat{x}\right)\right\}\\ \; & \overset{\left(b\right)}{\leq }C^{\pi^{\prime }}\left(x\right)+V_{\gamma }\left(\hat{x}\right), \end{array}$$

where (a) is due to (A14) and (12), (b) is due to γ∈(0,1). Recall the definition of $h_{\gamma }\left(x\right)$, by setting $M\left(x\right)=C^{\pi^{\prime }}\left(x\right)$, the condition (3) holds.

Based on Lemma A1, $M\left(x\right)=C^{\pi^{\prime }}\left(x\right)<\infty$ holds for any state x. Since there will be finite possible states after transition from x under any action, the sum of finite M(·) is also finite. Hence, condition (4) holds.

## Appendix D. Proof of Lemma 3

For (27), an equivalent transformation is made as follows:

(A17) V(Δ+1,q+1)+pV(Δ,q)≥V(Δ,q+1)+pV(Δ+1,q).

For every state x, we have

(A18) $$\begin{array}{cc} V(x) & =\min_{a\in A}Q\left(x,a\right)=\min\left\{Q(x,0),Q(x,1)\right\}. \end{array}$$

So every value function in (A17) has two possible values. In order to prove Equation (A17), theoretically we need to discuss $2^{4}=16$ cases, which is obviously a bit too cumbersome. Here we use a little trick, that is, as long as we prove that for the $2^{2}=4$ possible combinations on the left hand side(LHS) of (A17), there exists a combination on the right hand side (RHS) of (A17) to make “≥” hold, then we complete the proof. For convenience, we make a mapping by using four numbers to sequentially represent the action taken by the minimum state–action value function in Equation (A17). For example, “1010” represents the following:

(A19) $$\begin{array}{c} Q(\Delta +1,q+1,1)+pQ(\Delta ,q,0)\geq Q(\Delta ,q+1,1)+pQ(\Delta +1,q,0), \end{array}$$

So according to the previous trick, we only need to verify “0000”, “1010”, “0101”, and “1111” to prove Equation (A17). For brevity, we only show the verification process of “1010” in the following proof. The other three cases can also be proved by exactly the same steps.

Now we start to apply VIA and mathematical induction. Assuming that $V_{0}\left(x\right)=0$ for any states x, it is easy to yield

(A20) $$V_{1}\left(\Delta +1,q+1\right)+pV_{1}\left(\Delta ,q\right)\geq V_{1}\left(\Delta ,q+1\right)+pV_{1}\left(\Delta +1,q\right),$$

for any $q\in \left\{0,1,...,B-1\right\}$ and $\Delta \in Z^{+}$. By induction, assuming that for any $q\in \left\{0,1,\cdots ,B-1\right\}$ and $\Delta \in Z^{+}$, we have:

(A21) $$V_{k}\left(\Delta +1,q+1\right)+pV_{k}\left(\Delta ,q\right)\geq V_{k}\left(\Delta ,q+1\right)+pV_{k}\left(\Delta +1,q\right).$$

What we need to do is to verify that Equation (A21) still holds in the next value iteration. Based on our previous analysis, we will focus on the “1010” case. For $\Delta \in Z^{+}$ and $q\in \left\{0,1,\cdots ,B-1\right\}$, we have

(A22) $$\begin{array}{cc} \; & Q_{k}\left(\Delta +1,q+1,1\right)+pQ_{k}\left(\Delta ,q,0\right)\\ \; & -[Q_{k}\left(\Delta ,q+1,1\right)+pQ_{k}\left(\Delta +1,q,0\right)]\\ = & \Delta +1+p\lambda V_{k}\left(\Delta +2,q+1\right)+p\left(1-\lambda \right)V_{k}\left(\Delta +2,q\right)\\ \; & +\left(1-p\right)\lambda V_{k}\left(1,q+1\right)+\left(1-p\right)\left(1-\lambda \right)V_{k}\left(1,q\right)\\ \; & +p[\Delta +\omega C_{r}+\lambda V_{k}\left(\Delta +1,q+1\right)+\left(1-\lambda \right)V_{k}\left(\Delta +1,q\right)]\\ \; & -\Delta -p\lambda V_{k}\left(\Delta +1,q+1\right)-p\left(1-\lambda \right)V_{k}\left(\Delta +1,q\right)\\ \; & -\left(1-p\right)\lambda V_{k}\left(1,q+1\right)-\left(1-p\right)\left(1-\lambda \right)V_{k}\left(1,q\right)\\ \; & -p[\Delta +1+\omega C_{r}+\lambda V_{k}\left(\Delta +2,q+1\right)-\left(1-\lambda \right)V_{k}\left(\Delta +2,q\right)]\\ = & 1-p\geq 0. \end{array}$$

Therefore, by the similar step, we can verify the other three cases and confirm that the following formula

(A23) $$V_{k+1}\left(\Delta +1,q+1\right)+pV_{k+1}\left(\Delta ,q\right)\geq V_{k+1}\left(\Delta ,q+1\right)+pV_{k+1}\left(\Delta +1,q\right)$$

holds for any $\Delta \in Z^{+}$ and $q\in \left\{0,1,\cdots ,B-1\right\}$. Therefore, by induction, we prove that for any *k*, the Equation (A21) holds. Take the limits of *k* on both sides, then we are able to prove that (A17) holds, which indicates that (27) holds. Therefore, we have completed the proof.

## Appendix E. Proof of Theorem 2

By Corollary 1, the optimal policy is of a threshold structure if Q(x,a) has a *sub-modular* structure, that is,

(A24) Q(Δ,q,0)−Q(Δ,q,1)≤Q(Δ+1,q,0)−Q(Δ+1,q,1).

We will divide the whole proof into the following three cases:

**Case 1.** When q=0, for any $\Delta \in Z^{+}$ we have:

(A25) $$\begin{array}{cc} \; & Q(\Delta ,q,0)-Q(\Delta ,q,1)\\ = & \Delta +\lambda V(\Delta +1,q+1)+(1-\lambda )V(\Delta +1,q)\\ \; & -\Delta -\omega C_{r}-p\lambda V\left(\Delta +1,q+1\right)+p\left(1-\lambda \right)V\left(\Delta +1,q\right)\\ \; & -(1-p)\lambda V(1,q+1)-(1-p)(1-\lambda )V(1,q)\\ = & \left(1-p)\lambda (V(\Delta +1,q+1)-V(1,q+1)\right)\\ \; & +\left(1-p\right)\left(1-\lambda \right)\left(V\left(\Delta +1,q\right)-V\left(1,q\right)\right)-\omega C_{r}. \end{array}$$

Therefore, we have

(A26) $$\begin{array}{cc} \; & Q(\Delta +1,q,0)-Q(\Delta +1,q,1)-[Q(\Delta ,q,0)-Q(\Delta ,q,1)]\\ = & \left(1-p)\lambda (V(\Delta +2,q+1)-V(\Delta +1,q+1)\right)\\ \; & +(1-p)(1-\lambda )(V(\Delta +2,q)-V(\Delta ,q))\\ \overset{\left(a\right)}{\geq } & 0, \end{array}$$

where the last inequality (a) is due to (24) in Lemma 2.

**Case 2.** When $q\in \left\{1,\cdots ,B-1\right\}$, for any $\Delta \in Z^{+}$ we have

(A27) $$\begin{array}{cc} \; & Q(\Delta +1,q,0)-Q(\Delta +1,q,1)-[Q(\Delta ,q,0)-Q(\Delta ,q,1)]\\ = & Q(\Delta +1,q,0)-Q(\Delta ,q,0)-[Q(\Delta +1,q,1)-Q(\Delta ,q,1)]\\ = & \lambda [V(\Delta +2,q+1)-V(\Delta +1,q+1)]\\ \; & -p\lambda [V(\Delta +2,q)-V(\Delta +1,q)]\\ \; & +(1-\lambda )[V(\Delta +2,q)-V(\Delta +1,q)]\\ \; & -p(1-\lambda )[V(\Delta +2,q-1)-V(\Delta +1,q-1)]\\ \overset{\left(a\right)}{\geq } & 0, \end{array}$$

where the last inequality (a) is due to (27) in Lemma 3.

**Case 3.** When q=B, for any $\Delta \in Z^{+}$ we have

(A28) $$\begin{array}{cc} \; & Q(\Delta +1,q,0)-Q(\Delta +1,q,1)-[Q(\Delta ,q,0)-Q(\Delta ,q,1)]\\ = & Q(\Delta +1,q,0)-Q(\Delta ,q,0)-[Q(\Delta +1,q,1)-Q(\Delta ,q,1)]\\ = & \left(1-\lambda )[V(\Delta +2,q)-V(\Delta +1,q)\right]\\ \; & -p(1-\lambda )[V(\Delta +2,q-1)-V(\Delta +1,q-1)]\\ \overset{\left(a\right)}{\geq } & 0, \end{array}$$

where the last inequality (a) is also due to (27) in Lemma 3.

Therefore, we have completed the whole proof.

## Appendix F. Proof of Lemma A1

Before dealing with the expected discounted cost $C^{\pi^{\prime }}\left(x\right)$, we need to find the probability distribution of the first hitting time *T*, which is determined by the transition probabilities of system states. Under the lazy policy $\pi^{\prime }$, we can formulate a two-dimensional Markov chain to describe the dynamic changes of system states. The state transition probabilities of the formulated Markov chain is extremely complicated, and we can simplify it by combining some states, as depicted in Figure A1.

According to the simplified Markov chain, the initial state x can be divided into three cases: (☆,B), (·,B−1), and (·,q) where q<B−1. Note that for the special case $x=\hat{x}$, $C^{\pi^{\prime }}\left(x\right)$ is set to be 0. First, we focus on the case x=(·,q), where q<B−1. Suppose it takes T=k time slots for state x to transit to state $\hat{x}$ for the first time. Then state $x^{\prime }=\left(\cdot ,B-1\right)$ must be passed during these *k* time slots. Therefore, we can divide the entire transition process into two parts: state x first visits state $x^{\prime }$ after $k_{1}$ time slots, and then starts from state $x^{\prime }$ and enters state $\hat{x}$ for the first time after $k_{2}=k-k_{1}$ time slots. Denote $f_{x_{1},x_{2}}^{\left(n\right)}$ as the first hitting probability from state $x_{1}$ to state $x_{2}$ after *n* time slots, then we have

(A29) $$f_{x,\hat{x}}^{\left(k\right)}=\sum_{k_{1}=0}^{k}f_{x,x^{\prime }}^{\left(k_{1}\right)}f_{x^{\prime },\hat{x}}^{\left(k_{2}\right)}.$$

When the initial state first transits to state $x^{\prime }$, the total energy arrivals must be exactly B−q−1. Hence, the first hitting probability $f_{x,x^{\prime }}^{\left(k_{1}\right)}$ from state x to state $x^{\prime }$ can be expressed as follows:

(A30) fx,x′(k1)=k1−1B−q−2λB−q−2(1−λ)k1−1−(B−q−2)λ=k1−1B−q−2(λ1−λ)B−q−1(1−λ)k1≤(a)(k1−1)B−q−2(λ1−λ)B−q−1(1−λ)k1,

where $k_{1}\geq B-q-1$. The inequality (a) in (A30) is due to combination Nr≤Nr,∀N≥r. For any $k_{1}<B-q-1$, we have $f_{xx^{\prime }}^{\left(k_{1}\right)}=0$.

After entering state $x^{\prime }$, the system state will always change between states $x^{\prime }=\left(\cdot ,B-1\right)$ and (☆,B) before entering state $\hat{x}$ for the first time. By mathematical induction, $f_{x^{\prime },\hat{x}}^{\left(k_{2}\right)}$ is given as follows:

(A31) $$\begin{array}{cc} f_{x^{\prime },\hat{x}}^{\left(k_{2}\right)} & =\left[\begin{array}{cc} 1-\lambda & \lambda \end{array}\right]{\left[\begin{array}{cc} 1-\lambda & \lambda \\ 1-\lambda & \lambda p \end{array}\right]}^{k_{2}-2}\left[\begin{array}{c} 0\\ \lambda (1-p) \end{array}\right]\\ \; & =\left(1-p\right)\lambda^{2}\frac{\beta_{1}^{k_{2}-1}-\beta_{2}^{k_{2}-1}}{\beta_{1}-\beta_{2}}\\ \; & =\left(1-p\right)\lambda^{2}\sum_{i=0}^{k_{2}-2}\beta_{1}^{i}\beta_{2}^{k_{2}-2-i}\\ \; & \overset{\left(a\right)}{<}\left(1-p\right)\lambda^{2}\left(k_{2}-1\right)\beta_{1}^{k_{2}-2}, \end{array}$$

where $k_{2}\geq 2$, $\beta_{1}$ and $\beta_{2}$ are the eigenvalues of the matrix $\left[\begin{array}{cc} 1-\lambda & \lambda \\ 1-\lambda & \lambda p \end{array}\right]$ and satisfy $-1<\beta_{2}<0<1-\lambda <\beta_{1}<1$. The last inequality (a) of (A31) is due to $\beta_{2}<0<\beta_{1}$ and $\left|\beta_{2}\right|<\left|\beta_{1}\right|$. For any $k_{2}<2$, we have $f_{x^{\prime },\hat{x}}^{\left(k_{2}\right)}=0$.

Therefore, we will verify the discounted cost from the initial state x to reference state $\hat{x}$ is finite as follows:

(A32) $$\begin{array}{cc} C^{\pi^{\prime }}\left(x\right) & =E_{\pi^{\prime }}\left\{\sum_{t=0}^{T-1}\gamma^{t}C\left(x\left[t\right],a\left[t\right]\right)|x\left[0\right]=x\right\}\\ \; & \overset{\left(a\right)}{\leq }\sum_{k=0}^{\infty }f_{x,\hat{x}}^{\left(k\right)}\left[\sum_{t=0}^{k}\left(\Delta +t+\omega C_{r}\right)\right]\\ \; & \overset{\left(b\right)}{=}\sum_{k=B-q+1}^{\infty }\sum_{k_{1}=B-q-1}^{k}f_{x,x^{\prime }}^{\left(k_{1}\right)}f_{x^{\prime },\hat{x}}^{\left(k_{2}\right)}\left[\sum_{t=0}^{k}\left(\Delta +t+\omega C_{r}\right)\right]\\ \; & \overset{\left(c\right)}{\leq }\left(1-p\right)\lambda^{2}\frac{{\left(\frac{1-\lambda }{\beta_{1}}\right)}^{B-q-1}}{1-\frac{1-\lambda }{\beta_{1}}}\sum_{k=2}^{\infty }\beta_{1}^{k-2}k^{B-q-1}\left[\sum_{t=0}^{k}\left(\Delta +t+\omega C_{r}\right)\right]\\ \; & \overset{\left(d\right)}{<}\infty . \end{array}$$

where inequality (a) is due to (A11), equality (b) is due to (A29), inequality (c) is due to (A30) and (A31), and inequality (d) is due to $0<\beta_{1}<1$.

For the other two case where the initial state is (·,B−1) or (☆,B), the discounted cost to the reference state for the first time can also be verified to be finite by similar steps. Therefore, we have completed the proof of Lemma A1.
